# Antihypertensive Regimens in Stabilized Hypertensive Patients at a Malagasy Tertiary Hospital

**DOI:** 10.7759/cureus.111261

**Published:** 2026-06-21

**Authors:** Rado Olivier Rakoto Sedson, Holy Mihanta Sabrina Ranaivoson, Rija Mikhael Miandrisoa, Mamisoa Herinirina Nomenjanahary, Marie Osé Judicaël Harioly Nirina, Solofonirina Rakotoarimanana, Nirina Rabearivony

**Affiliations:** 1 Faculty of Medicine, University of Toamasina, Toamasina, MDG; 2 Faculty of Medicine, University of Fianarantsoa, Fianarantsoa, MDG; 3 Faculty of Medicine, University of Antananarivo, Antananarivo, MDG; 4 Faculty of Medicine, University of Antsinanana, Antsiranana, MDG

**Keywords:** antihypertensive therapy, cardiovascular risk, hypertension, madagascar, polytherapy, sub-saharan africa, target organ damage

## Abstract

Introduction

Hypertension remains a major public health challenge globally, with increasing prevalence in sub-Saharan Africa. Achieving blood pressure control often requires combination antihypertensive therapy, yet data on treatment patterns in African settings remain limited. This study aimed to describe antihypertensive treatment regimens among stabilized hypertensive patients at a tertiary care center in Madagascar.

Methodology

A descriptive cross-sectional study was conducted at the Cardiology Department of University Hospital Morafeno Toamasina from June 30 to December 30, 2025. Consecutive sampling included patients aged ≥18 years with controlled hypertension (systolic blood pressure <140 mmHg and/or diastolic blood pressure <90 mmHg) on antihypertensive therapy for ≥3 months. Data on sociodemographic characteristics, clinical variables, cardiovascular risk factors, target organ complications, and treatment regimens were collected. Bivariate analysis with odds ratios (OR) and 95% confidence intervals (CI) was performed to identify factors associated with different treatment patterns.

Results

Among 126 stabilized hypertensive patients, polytherapy predominated, with 122 patients (96.83%) receiving combination therapy. Bitherapy was prescribed to 56 (44.44%) patients, tritherapy to 51 (40.48%) patients, quadritherapy to 15 (11.90%) patients, and monotherapy to only 4 (3.17%) patients. Calcium channel blockers (CCBs) were the most frequently prescribed drug class, used in 98 (77.8%) patients, followed by angiotensin receptor blockers (ARBs) in 88 (69.8%) patients and thiazide diuretics in 65 patients (51.6%). The ARB + CCB combination was the dominant bitherapy regimen, representing 32 (57.1%) of 56 bitherapy patients, while the renin-angiotensin system (RAS) blocker + CCB + thiazide backbone accounted for 29 (56.9%) of 51 tritherapy patients. Significant associations included cardiac complications with quadritherapy (OR=12.88, 95% CI: 3.61-45.89, p<0.001), high cardiovascular risk with quadritherapy (OR=4.47, 95% CI: 1.42-14.08, p=0.009) and tritherapy (OR=2.44, 95% CI: 1.15-5.18, p=0.023), absence of cardiac complications with bitherapy (OR=0.08, 95% CI: 0.01-0.63, p=0.003), absence of neurological complications with bitherapy (OR=0.31, 95% CI: 0.10-0.99, p=0.044), and absence of high cardiovascular risk with bitherapy (OR=0.19, 95% CI: 0.08-0.45, p<0.001).

Conclusions

This study demonstrates a rational, risk-stratified approach to antihypertensive therapy at University Hospital Morafeno Toamasina, with treatment intensity guided by cardiovascular risk and target organ complications. Cardiac complications emerged as the strongest predictor of treatment intensification. These findings provide valuable baseline data for quality improvement initiatives and highlight the feasibility of guideline-concordant hypertension management in resource-limited African settings.

## Introduction

Hypertension constitutes the leading modifiable risk factor for cardiovascular disease, stroke, and chronic kidney disease worldwide, affecting over one billion individuals globally [1]. The burden of hypertension is particularly pronounced in sub-Saharan Africa; in 2014, the prevalence rates increased dramatically due to epidemiological transition, urbanization, and lifestyle modifications [2]. In this region, hypertension is frequently diagnosed at advanced stages with established target organ damage, necessitating intensive therapeutic interventions, a pattern that underscores the importance of the risk-stratified approach outlined in the 2024 European Society of Cardiology (ESC) Guidelines for the management of elevated blood pressure and hypertension [3].

Contemporary international guidelines include the 2024 ESC guidelines for the management of elevated blood pressure and hypertension [3] and the 2025 American College of Cardiology/American Heart Association (ACC/AHA) guidelines [4]. However, the translation of these evidence-based recommendations into clinical practice varies considerably across different healthcare settings and geographical regions [5].

Despite the growing burden of hypertension in Africa, comprehensive data on real-world antihypertensive prescribing patterns in African tertiary care settings remain scarce. Madagascar, an island nation in the Indian Ocean region of sub-Saharan Africa, faces similar challenges to mainland African countries regarding hypertension management, including limited access to specialized care, economic constraints, and late disease presentation. Understanding current treatment practices in this context is essential for quality improvement initiatives, resource allocation, and adaptation of international guidelines to local realities.

This study aimed to describe antihypertensive treatment regimens among stabilized hypertensive patients at University Hospital Morafeno Toamasina, a tertiary referral center in Madagascar, to identify clinical factors associated with treatment intensification. We hypothesized that treatment patterns would reflect a risk-stratified approach consistent with international guidelines, with treatment complexity increasing in proportion to cardiovascular risk and target organ damage.

## Materials and methods

Study design and setting

This retrospective descriptive cross-sectional study was conducted at the Cardiology Department of University Hospital Morafeno Toamasina, a tertiary care university hospital in Toamasina, Madagascar. The study period extended from June 30 to December 30, 2025. The Cardiology Department serves as a regional referral center for cardiovascular diseases, providing specialized outpatient and inpatient services to a predominantly urban and peri-urban population.

Study population

The target population comprised adult hypertensive patients receiving antihypertensive therapy and followed in the outpatient cardiology clinic. Consecutive sampling was employed to recruit all eligible patients presenting during the study period.

Selection criteria

The inclusion and exclusion criteria are presented in Table 1.

**Table 1:** Inclusion and Exclusion Criteria

Criterion type	Criterion
Inclusion	Age ≥18 years
Inclusion	Established diagnosis of hypertension
Inclusion	Receiving antihypertensive therapy for ≥3 months
Inclusion	Stabilized blood pressure at recent consultations; systolic blood pressure <140 mmHg and/or diastolic blood pressure <90 mmHg at ≥2 consecutive visits (taking into account the blood pressure targets recommended in the guidelines [3,4])
Exclusion	Newly diagnosed hypertension (treatment duration <3 months)
Exclusion	Incomplete medical records
Exclusion	Gestational hypertension (pregnant women)

Operational definitions

Stabilized hypertensive patient: A patient with established hypertension receiving antihypertensive therapy who achieved blood pressure control, defined as systolic blood pressure <140 mmHg and/or diastolic blood pressure <90 mmHg, documented at a minimum of two consecutive outpatient visits, taking into account the blood pressure targets recommended in the guidelines [3,4].

High cardiovascular risk: Patients classified as having elevated global cardiovascular risk based on the presence of multiple cardiovascular risk factors, established cardiovascular disease, target organ damage, or diabetes mellitus.

Cardiac complications: Documented cardiac target organ damage, including left ventricular hypertrophy, heart failure, coronary artery disease, or other structural cardiac abnormalities.

Neurological complications: History of stroke, transient ischemic attack, or other cerebrovascular events.

Vascular complications: Peripheral arterial disease or other vascular manifestations of hypertension.

Renal complications: Chronic kidney disease or hypertensive nephropathy.

Variables included

Sociodemographic variables: Age, sex.

Clinical variables: Blood pressure measurements, comorbidities (diabetes mellitus, dyslipidemia), cardiovascular risk factors (smoking), target organ complications (cardiac, vascular, renal, neurological), and global cardiovascular risk stratification.

Therapeutic variables: Antihypertensive drug classes, number of medications, treatment regimen classification (monotherapy, bitherapy, tritherapy, quadritherapy), medication adherence, and adverse effects.

Statistical analysis

Data were analyzed using appropriate Epi Info™ version 7.x (Centers for Disease Control and Prevention, Atlanta, GA, USA). Descriptive statistics included frequencies and percentages for categorical variables. Bivariate analysis was performed to examine associations between treatment regimens and clinical characteristics. Crude odds ratios (ORs) with 95% confidence intervals (CIs) were calculated using the cross-tabulation method. Fisher’s exact test or chi-square test was employed for categorical variables, with statistical significance set at p <0.05.

Ethical considerations

The study protocol was approved by the institutional ethics committee of the University Hospital Morafeno and the Faculty of Medicine of Toamasina. As this was a retrospective study using anonymized data from medical records, the requirement for individual informed consent was waived. All data were handled in accordance with principles of medical confidentiality and data protection. Patient identifiers were removed from the database, and each patient was assigned a unique study identification number.

## Results

Study population characteristics

A total of 126 stabilized hypertensive patients were enrolled during the six-month study period. The cohort comprised patients with controlled blood pressure on stable antihypertensive regimens for a minimum of three months.

Age and sex distribution

The mean age of the study population was 58 years, with a range from 29 to 88 years. The age distribution revealed that the 61-70-year age group was the most represented, accounting for 35 (27.8%) patients, followed by the 51-60-year group (26.2%, n=64). The sex distribution showed a predominance of women, yielding a sex ratio (male:female) of 0.50.

Cardiovascular risk factors

Dyslipidemia was the most prevalent risk factor, affecting 28.6% (n=36) of patients, followed by diabetes mellitus (18.3%, n=23) and active smoking (0.8%, n=1). The number of patients classified as being at high cardiovascular risk was 44 (34.92%).

Target organ damage

Neurological complications were observed in 18 (14.3%) patients, followed by cardiac complications (11.1%, n=14), vascular complications (2.3%, n=3), and renal complications (0.8%, n=1).

Distribution of antihypertensive treatment regimens

The distribution of treatment regimens revealed a marked predominance of combination therapy. Only 4 (3.17%) patients received monotherapy, while 122 (96.83%) patients were treated with polytherapy. Among polytherapy regimens, bitherapy was the most common, prescribed to 56 (44.44%) patients, followed by tritherapy in 51 (40.48%) patients and quadritherapy in 15 (11.90%) patients.

Antihypertensive drug classes and combination patterns 

Drug combination patterns by treatment regimen are shown in Table 2. Calcium channel blockers (CCBs) were the most frequently prescribed drug class, used in 98 (77.8%) patients, followed by angiotensin receptor blockers (ARBs) in 88 (69.8%) patients and thiazide diuretics in 65 (51.6%) patients. The most frequently prescribed bitherapy combination was ARB + CCB, representing 32 (57.1%) patients of dual-drug regimens. All quadritherapy regimens included a renin-angiotensin system (RAS) blocker (angiotensin-converting enzyme inhibitor (ACEI) or ARB) combined with a CCB, with the addition of a thiazide diuretic, beta-blocker, or aldosterone antagonist as the fourth agent.

**Table 2:** Drug Combination Patterns by Treatment Regimen (n=126) ARBs: angiotensin receptor blockers; ACEI: angiotensin-converting enzyme inhibitors.

Drug combination	n	%
Bitherapy (n=56)		
ARB + Calcium channel blocker	32	57.1
ACEI + Thiazide diuretic	8	14.3
ACEI + Calcium channel blocker	8	14.3
ARB + Thiazide diuretic	6	10.7
ARB + Aldosterone antagonist	1	1.8
ACEI + Beta-blocker	1	1.8
Tritherapy (n=51)		
ARB + Thiazide + Calcium channel blocker	23	45.1
ARB + Calcium channel blocker + Beta-blocker	7	13.7
ACEI + Thiazide + Calcium channel blocker	6	11.8
ACEI + Calcium channel blocker + Beta-blocker	5	9.8
ACEI + Thiazide + Beta-blocker	4	7.8
ARB + Thiazide + Beta-blocker	2	3.9
ARB + Thiazide + Aldosterone antagonist	3	5.9
ARB + Calcium channel blocker + Aldosterone antagonist	1	2.0
Quadritherapy (n=15)		
ARB + Thiazide + Calcium channel blocker + Beta-blocker	5	33.3
ACEI + Thiazide + Calcium channel blocker + Aldosterone antagonist	2	13.3
ACEI + Thiazide + Calcium channel blocker + Beta-blocker	2	13.3
ARB + Calcium channel blocker + Beta-blocker + Aldosterone antagonist	2	13.3
ARB + Thiazide + Calcium channel blocker + Aldosterone antagonist	1	6.7
ARB + Thiazide + Calcium channel blocker + Central agent	1	6.7
ARB + Calcium channel blocker + Beta-blocker + Central agent	1	6.7
ACEI + Thiazide + Aldosterone antagonist + Beta-blocker	1	6.7

Adverse effects

Among the adverse effects of medication, cough was reported in 3.2% of cases and lower limb edema in 12.7%.

Factors associated with monotherapy 

No statistically significant associations were identified between monotherapy and any of the examined variables.

Factors associated with bitherapy 

The factors associated with bitherapy are summarized in Table 3. Three statistically significant inverse associations were identified for bitherapy: absence of cardiac complications (OR=0.08, 95% CI: 0.01-0.63, p=0.003), absence of neurological complications (OR=0.31, 95% CI: 0.10-0.99, p=0.044), and absence of high cardiovascular risk (OR=0.19, 95% CI: 0.08-0.45, p<0.001).

**Table 3:** Factors Associated With Bitherapy (n=126) *Statistically significant (p<0.05). OR: odds ratio; CI: confidence interval.

Variable	Bitherapy		OR	95% CI	p-Value
	Yes, n (%)	No, n (%)			
Age >65 years					
Yes	41 (47.67)	45 (52.33)	1.52	0.70-3.27	0.338
No	15 (37.50)	25 (62.50)			
Sex					
Female	37 (45.12)	45 (54.88)	1.08	0.51-2.26	0.853
Male	19 (43.18)	25 (56.82)			
Diabetes mellitus					
Yes	8 (34.78)	15 (65.22)	0.61	0.24-1.57	0.358
No	48 (46.60)	55 (53.40)			
Dyslipidemia					
Yes	20 (55.56)	16 (44.44)	1.88	0.86-4.09	0.119
No	36 (40.00)	54 (60.00)			
Smoking					
Yes	1 (100)	0 (0)	Undefined	Undefined	0.444
No	55 (44.00)	70 (56.00)			
Cardiac complications					
Yes	1 (7.14)	13 (92.86)	0.08	0.01-0.63	0.003*
No	55 (49.11)	57 (50.89)			
Vascular complications					
Yes	1 (33.33)	2 (66.67)	0.62	0.01-12.21	1.000
No	55 (44.72)	68 (55.28)			
Renal complications					
Yes	0 (0)	1 (100)	0.00	Undefined	1.000
No	56 (44.80)	69 (55.20)			
Neurological complications					
Yes	4 (22.22)	14 (77.78)	0.31	0.10-0.99	0.044*
No	52 (48.15)	56 (51.85)			
High cardiovascular risk					
Yes	9 (20.45)	35 (79.55)	0.19	0.08-0.45	<0.001*
No	45 (57.32)	35 (42.68)			
Total	56 (44.44)	70 (55.56)	-	-	-

Factors associated with tritherapy 

One statistically significant positive association was identified for tritherapy: high cardiovascular risk (OR=2.44, 95% CI: 1.15-5.18, p=0.023). The factors associated with tritherapy are presented in Table 4.

**Table 4:** Factors Associated With Tritherapy (n=126) *Statistically significant (p<0.05). OR: odds ratio; CI: confidence interval.

Variable	Tritherapy		OR	95% CI	p-Value
	Yes, n (%)	No, n (%)			
Age >65 years					
Yes	34 (39.53)	52 (60.47)	0.88	0.41-1.89	0.846
No	17 (42.50)	23 (57.50)			
Sex					
Female	35 (42.68)	47 (57.32)	1.30	0.61-2.77	0.570
Male	16 (36.36)	28 (63.64)			
Diabetes mellitus					
Yes	11 (47.83)	12 (52.17)	1.44	0.58-3.58	0.485
No	40 (38.83)	63 (61.17)			
Dyslipidemia					
Yes	11 (30.56)	25 (69.44)	0.55	0.24-1.25	0.166
No	40 (44.44)	50 (55.56)			
Smoking					
Yes	0 (0)	1 (100)	0.00	Undefined	1.000
No	51 (40.80)	74 (59.20)			
Cardiac complications					
Yes	6 (42.86)	8 (57.14)	1.12	0.36-3.44	1.000
No	45 (40.18)	67 (59.82)			
Vascular complications					
Yes	1 (33.33)	2 (66.67)	0.73	0.06-8.27	1.000
No	50 (40.65)	73 (59.35)			
Renal complications					
Yes	0 (0)	1 (100)	0.00	Undefined	1.000
No	51 (40.80)	74 (59.20)			
Neurological complications					
Yes	10 (55.56)	8 (44.44)	2.04	0.75-5.60	0.197
No	41 (37.96)	67 (62.04)			
High cardiovascular risk					
Yes	24 (54.55)	20 (45.45)	2.44	1.15-5.18	0.023*
No	27 (32.93)	55 (67.07)			
Total	51 (40.48)	75 (59.52)	-	-	-

Factors associated with quadritherapy

Two statistically significant positive associations were identified for quadritherapy: cardiac complications (OR=12.88, 95% CI: 3.61-45.89, p<0.001) and high cardiovascular risk (OR=4.47, 95% CI: 1.42-14.08, p=0.009). Table 5 shows the distribution of factors associated with quadritherapy.

**Table 5:** Factors Associated With Quadritherapy (n=126) *Statistically significant (p<0.05). OR: odds ratio; CI: confidence interval.

Variable	Quadritherapy		OR	95% CI	p-Value
	Yes, n (%)	No, n (%)			
Age >65 years					
Yes	8 (9.41)	77 (90.59)	0.49	0.16-1.46	0.240
No	7 (17.50)	33 (82.50)			
Sex					
Female	7 (8.64)	74 (91.36)	0.43	0.14-1.27	0.151
Male	8 (18.18)	36 (81.82)			
Diabetes mellitus					
Yes	4 (17.39)	19 (82.61)	1.74	0.50-6.06	0.475
No	11 (10.78)	92 (89.22)			
Dyslipidemia					
Yes	5 (13.89)	31 (86.11)	1.27	0.40-4.02	0.763
No	10 (11.24)	80 (88.76)			
Smoking					
Yes	0 (0)	1 (100)	0.00	Undefined	1.000
No	15 (12.10)	110 (87.90)			
Cardiac complications					
Yes	7 (50.00)	7 (50.00)	12.88	3.61-45.89	<0.001*
No	8 (7.21)	104 (92.79)			
Vascular complications					
Yes	1 (33.33)	2 (66.67)	3.86	0.33-45.34	0.321
No	14 (11.48)	109 (88.52)			
Renal complications					
Yes	1 (100)	0 (0)	Undefined	Undefined	0.120
No	14 (11.29)	111 (88.71)			
Neurological complications					
Yes	3 (16.67)	15 (83.33)	1.58	0.40-6.28	0.453
No	12 (11.21)	96 (88.79)			
High cardiovascular risk					
Yes	10 (22.73)	34 (77.27)	4.47	1.42-14.08	0.009*
No	5 (6.17)	77 (93.83)			
Total	15 (11.90)	111 (88.10)	-	-	-

Summary of treatment stratification

The analysis revealed a clear therapeutic gradient based on cardiovascular risk and target organ complications: bitherapy (44.44%, n= 56) was preferentially used in patients without target organ complications and at lower cardiovascular risk, tritherapy (40.48%, n=51) was associated with high cardiovascular risk in the absence of established cardiac complications, and quadritherapy (11.90%, n=15) was strongly associated with cardiac complications and very high cardiovascular risk.

## Discussion

Principal findings

This cross-sectional study of 126 stabilized hypertensive patients at University Hospital Morafeno Toamasina revealed several key findings. First, combination antihypertensive therapy predominated overwhelmingly, with 122 patients receiving polytherapy and only four patients on monotherapy, a finding consistent with the well-established evidence that monotherapy rarely achieves adequate blood pressure control in patients with established hypertension, particularly in specialist cardiology settings where case complexity is high [1,2]. Second, bitherapy and tritherapy were nearly equally distributed, with quadritherapy required in 15 patients. Third, a clear therapeutic gradient emerged, with treatment intensity stratified according to cardiovascular risk and target organ damage, in line with the fundamental principle endorsed by both ESC/ESH and ACC/AHA guidelines that treatment intensity should be proportional to global cardiovascular risk [3,4]. Fourth, cardiac complications emerged as the strongest predictor of treatment intensification to quadritherapy, while the absence of complications and lower cardiovascular risk were associated with bitherapy. These findings demonstrate a rational, guideline-concordant approach to hypertension management in this tertiary-care African setting, comparable to what has been reported in other sub-Saharan African specialist centers [5,6].

Comparison with African, Asian, and Western literature

The treatment patterns documented at University Hospital Morafeno Toamasina are broadly consistent with data from specialized centers across sub-Saharan Africa, Asia, and Western countries, while reflecting the particular complexity of a tertiary cardiology population. In the African context, a systematic review and meta-analysis of antihypertensive treatment across sub-Saharan Africa reported monotherapy in 21%, bitherapy in 42.6%, and tritherapy in 26.6% of patients [7]; our markedly lower monotherapy rate and higher tritherapy rate indicate a more severe case-mix, a finding corroborated by evidence from nephrology and cardiology centers in Côte d’Ivoire and Nigeria, where the presence of target organ damage was consistently associated with more intensive combination regimens [8], and by a recent initiative in rural sub-Saharan Africa that confirmed the feasibility of single-pill triple therapy even in resource-limited settings [9]. Notably, a study specifically conducted in Madagascar and Mauritius reported that a fixed-dose triple combination (perindopril/indapamide/amlodipine) effectively controlled blood pressure in a sub-Saharan African island population, providing direct regional support for the intensive polytherapy approach observed in our cohort [10]. In Asian populations, a study of Chinese diabetic patients reported monotherapy in 42.98% and polytherapy in 57.02%, a substantially lower polytherapy burden than our cohort, likely reflecting a less complex patient profile [11]; a large multinational analysis published in JAMA Network Open further demonstrated that RAS inhibitors combined with CCBs or diuretics constitute the preferred dual escalation strategy across the region [12], a pattern that our high bitherapy rate suggests is similarly applied at Toamasina, while evidence from a tertiary center in Saudi Arabia confirmed that guideline-concordant combination therapy is achievable in diverse low- and middle-income settings [13]. In Western countries, a Polish hospital cohort reported that 63.7% of hypertensive inpatients received three or more antihypertensive drugs [14], a benchmark our study approaches with 52.38% of patients on tritherapy or quadritherapy; randomized trials of triple fixed-dose combinations have demonstrated superior efficacy over dual therapy for stage 2 hypertension [15], supporting the clinical rationale for the intensive regimens observed in our cohort. Across all three regions, the fundamental drivers of treatment intensification, target organ damage, particularly cardiac complications, and elevated global cardiovascular risk, remain consistent, underscoring that the principles of risk-stratified hypertension management transcend geographical and economic boundaries.

Antihypertensive drug classes and combinations: Local practice and international context

The drug class profile observed at University Hospital Morafeno Toamasina is characterized by the predominance of CCBs, ARBs, and thiazide diuretics, with ACE inhibitors (ACEIs) prescribed in 29.4% of patients, beta-blockers in 24.6%, aldosterone antagonists (spironolactone) in 9.5%, and central-acting agents in only 1.6%. This pharmacological profile is broadly consistent with international prescribing patterns in sub-Saharan Africa. The multi-country EIGHT study conducted across 12 African nations reported that CCBs and diuretics were the most frequently used antihypertensive classes, with amlodipine and hydrochlorothiazide among the top molecules, while RAS blockers (ACEIs and ARBs) featured prominently across all settings [16]. Similarly, studies from Nigeria and Ethiopia documented the dominance of CCBs and thiazide diuretics in both primary and tertiary care settings, with atenolol and lisinopril/losartan as the most commonly named individual agents [17]. The near-universal use of RAS blockade at CHU Morafeno Toamasina reflects the strong evidence base supporting RAS inhibition in hypertensive patients with cardiovascular risk factors, diabetes, and target organ damage, as endorsed by both ESC/ESH and ACC/AHA guidelines [3,4].

The predominance of ARBs over ACEIs in our cohort aligns with prescribing trends observed across sub-Saharan Africa, where ARBs are increasingly preferred due to their superior tolerability profile, particularly the absence of ACEI-associated cough, which is more prevalent in African populations [17,18]. A comparative effectiveness analysis in the UK Clinical Practice Research Datalink further confirmed that ARBs and ACEIs differ in tolerability profiles across ethnic groups, with ARBs offering a more favorable side-effect profile in non-White populations [18]. International guidelines for people of African descent also emphasize CCBs and diuretics as particularly effective first-line options due to the low-renin hypertension phenotype more prevalent in Black patients, consistent with the high CCB usage observed in our study [19,20].

The combination patterns further reflect guideline adherence. In bitherapy, the ARB + CCB combination was dominant, followed by ACEI/ARB + thiazide, consistent with the preferred dual combinations recommended by ESC/ESH guidelines (RAS blocker + CCB or diuretic) and corroborated by the JAMA Network Open multinational analysis showing RAS + CCB as the most effective dual strategy [12]. In tritherapy, the combination RAS blocker + CCB + thiazide diuretic accounted for 56.9% of three-drug regimens, precisely mirroring the guideline-recommended triple backbone for resistant hypertension [3,4]. The addition of beta-blockers in quadritherapy regimens is consistent with their established role in hypertension complicated by heart failure with reduced ejection fraction or stable coronary artery disease, as supported by multiple randomized trials and meta-analyses demonstrating mortality benefit in these specific cardiac comorbidities [21]. The use of aldosterone antagonists (spironolactone) in quadritherapy is supported by the landmark PATHWAY-2 randomized crossover trial, which demonstrated that spironolactone was superior to bisoprolol and doxazosin as fourth-line therapy for drug-resistant hypertension [22], a finding endorsed by current ESC/ESH and ACC/AHA guidelines as the preferred add-on agent in this context [3,4]. The limited use of central-acting agents reflects their relegation to last-resort status in contemporary guidelines, appropriate in a stabilized specialist cohort. Overall, the pharmacological profile at University Toamasina Morafeno Toamasina demonstrates a high degree of alignment with evidence-based prescribing principles, comparable to patterns reported from specialized centers across Africa, Asia, and Western countries.

Risk-stratified treatment intensification

A key finding of our study is the clear therapeutic gradient based on cardiovascular risk and target organ complications. Patients without complications and at lower cardiovascular risk predominantly received bitherapy. Patients at high cardiovascular risk without established cardiac complications were more likely to receive tritherapy. Patients with cardiac complications were strongly associated with quadritherapy. This stratification pattern is entirely consistent with contemporary guideline recommendations from the ESC/ESH and ACC/AHA, which advocate for treatment intensity proportional to cardiovascular risk and the presence of target organ damage [3,4]. The strong association between cardiac complications and quadritherapy indicates that clinicians at University Hospital Morafeno Toamasina appropriately recognize the need for intensive blood pressure control in patients with cardiac target organ damage, including left ventricular hypertrophy, heart failure, or coronary artery disease, conditions for which more intensive blood pressure lowering has been shown to reduce cardiovascular events and mortality [23]. Similarly, the inverse association between absence of complications and bitherapy demonstrates appropriate restraint in treatment intensification for lower-risk patients, a balanced approach that avoids unnecessary polypharmacy while ensuring optimal protection for high-risk individuals. This nuanced risk-guided prescribing is consistent with the approach recommended in the diagnosis and management of resistant hypertension literature, which emphasizes systematic assessment of cardiovascular risk burden before escalating therapy [24,25].

Clinical implications

The findings of this study have several important clinical implications. First, the predominance of combination therapy demonstrates that monotherapy is rarely sufficient to achieve blood pressure control in patients followed in specialized cardiology settings, supporting guideline recommendations for early initiation of combination therapy in most hypertensive patients. Second, the clear association between treatment intensity and cardiovascular risk validates the importance of comprehensive cardiovascular risk assessment, including systematic evaluation for target organ damage, in guiding treatment decisions, consistent with the pathophysiologically based prescribing approach advocated for sub-Saharan African populations. Third, the substantial proportion of patients requiring tritherapy or quadritherapy highlights the complexity of hypertension management in tertiary care settings, necessitating specialized expertise, regular follow-up, and attention to medication adherence, a recognized challenge in resource-limited settings where polypharmacy may compromise adherence [5,8,10,21]. Fourth, the convergence between our findings and international data demonstrates that guideline-concordant hypertension management is feasible in African tertiary care settings; the potential adoption of fixed-dose combination pills could further improve adherence and blood pressure control in this context, as suggested by recent proposals for low-dose triple-drug combination protocols in sub-Saharan Africa. However, efforts to improve hypertension control across Madagascar must also address healthcare access, medication availability, and affordability beyond specialized centers.

Strengths of the study

This study possesses several methodological strengths. The use of a clear operational definition for “stabilized hypertensive patient” ensures population homogeneity and facilitates international comparison. The requirement for a minimum treatment duration of three months ensures that observed regimens represent stable therapeutic strategies rather than transient adjustments, a methodological approach that distinguishes this study from cross-sectional analyses of newly diagnosed patients. The exhaustive consecutive sampling approach minimizes selection bias, while rigorous bivariate analysis with ORs and 95% CIs allows nuanced interpretation of associations. The study provides valuable contextual data from Madagascar, a country underrepresented in the international hypertension literature, and its direct comparison with African, Asian, and Western evidence contextualizes findings within the global evidence base. Finally, the focus on stabilized patients offers practical insights into effective treatment strategies that have successfully achieved blood pressure control, with direct applicability to quality improvement and medical education.

Limitations

Several limitations warrant consideration. The cross-sectional design precludes causal inference and does not capture the temporal evolution of treatment regimens or long-term outcomes; longitudinal studies would be needed to assess the impact of treatment intensification on cardiovascular events and mortality. The very small monotherapy group limited statistical power for that subgroup, as well as the number of smokers (n=1). Although drug class frequencies and combination patterns were documented, individual molecule-level data (e.g., specific ARB or ACEI molecules) were not systematically recorded, precluding comparison at the molecular level, an important limitation given the documented differences in prescribing patterns at the molecular level across African countries. Recruitment from a single specialized cardiology department limits generalizability to primary care or rural settings, and the six-month study period may not reflect seasonal prescribing variations.

## Conclusions

This study demonstrates the overwhelming predominance of combination therapy, reflecting appropriate recognition that most hypertensive patients require multiple agents to achieve blood pressure control. A clear therapeutic gradient was observed: bitherapy for lower-risk patients without complications, tritherapy for high-risk patients, and quadritherapy for those with established cardiac complications. CCBs and ARBs were the most widely prescribed drug classes, and combination patterns, dominated by ARB + CCB in and RAS blocker + CCB + thiazide in tritherapy, closely mirror guideline-recommended regimens. Cardiac complications emerged as the strongest predictor of treatment intensification, underscoring the importance of systematic screening for target organ damage in guiding therapeutic decisions. These findings that provide valuable baseline data for quality improvement initiatives contribute to the limited literature on hypertension management in Madagascar and sub-Saharan Africa, and demonstrate that evidence-based, guideline-concordant cardiovascular care is attainable in resource-limited settings; future research should employ longitudinal multicenter designs to assess treatment evolution, medication adherence, and outcomes across primary care and rural settings.
